# CD20 positive CD8 T cells are a unique and transcriptionally-distinct subset of T cells with distinct transmigration properties

**DOI:** 10.1038/s41598-021-00007-0

**Published:** 2021-10-15

**Authors:** Martijn Vlaming, Vrouyr Bilemjian, Jimena Álvarez Freile, Harm Jan Lourens, Nienke van Rooij, Gerwin Huls, Tom van Meerten, Marco de Bruyn, Edwin Bremer

**Affiliations:** 1 Department of Hematology, University Medical Center Groningen, University of Groningen, Groningen, The Netherlands; 2 Department of Obstetrics and Gynecology, University Medical Center Groningen, University of Groningen, Groningen, The Netherlands

**Keywords:** Cell adhesion, Cell migration

## Abstract

The presence of T cells that are dimly positive for the B cell marker CD20 is well-established in autoimmunity and correlates with disease severity in various diseases. Further, we previously identified that the level of CD20-positive T cells was three–fourfold elevated in ascites fluid of ovarian carcinoma patients, together suggesting a role in both autoimmunity and cancer. In this respect, treatment of autoimmune patients with the CD20-targeting antibody Rituximab has also been shown to target and deplete CD20-positive T cells, previously identified as IFN-gamma producing, low proliferative, CD8 cytotoxic T cells with an effector memory (EM) differentiation state. However, the exact phenotype and relevance of CD20-positive T cells remains unclear. Here, we set out to identify the transcriptomic profile of CD20-positive T cells using RNA sequencing. Further, to gain insight into potential functional properties of CD20 expression in T cells, CD20 was ectopically expressed on healthy human T cells and phenotypic, functional, migratory and adhesive properties were determined in vitro and in vivo. Together, these assays revealed a reduced transmigration and an enhanced adhesive profile combined with an enhanced activation status for CD20-positive T cells.

## Introduction

The tetraspanin cell surface protein CD20 is a prototypical B cell marker and is a prominent target for antibody-based therapy of B cell malignancies, such as non-Hodgkin’s lymphoma, as well as autoimmune diseases, such as rheumatoid arthritis (RA) and multiple sclerosis (MS)^1,2^. Treatment with CD20-targeting antibodies is curative in many B cell malignancies and ameliorates disease activity in several autoimmune diseases^2^.

Interestingly, the therapeutic effect of CD20 antibodies may be partly attributed to the targeting of a subpopulation of T cells that selectively expresses CD20^3,4^. Such T cells express CD20 at a level ~ 15 fold lower than B cells^5^, but do not express other hallmark B cell markers, such as CD19^6^. CD20-positive T cells are found in the peripheral blood of autoimmune and cancer patients, but also in healthy individuals at ~ 3–5% of the total CD3 T cell population^5^. Treatment with CD20 antibodies depletes such CD20-positive T cells in RA, MS, psoriasis and immune-mediated thrombotic thrombocytopenic purpura^3,4^. Interestingly, in RA, MS and psoriasis, these CD20-positive T cells correlated with disease severity and produced an increased level of pro-inflammatory cytokines^7–10^, suggesting that CD20-positive T cells may play a role in pathophysiology^11^. Correspondingly, it has been postulated that some of the clinical effects achieved by CD20-targeting antibodies in autoimmunity may be attributable to elimination of CD20-positive T cells^4,10^. Of note, CD20-positive T cells were also reported in HIV patients, where CD20 expression on CD4 T cells was reported to be enhanced during untreated HIV infection^12^. Upon antiretroviral therapy these cells were reported to contain high levels of HIV transcripts, making them ideal targets for Rituximab for reducing the transcriptionally active HIV-reservoir. This suggests a virus-specific effect on CD20 expression in T cells and CD20 as a marker for HIV-infected cells.

Studies in the context of cancer are limited, but we previously reported that CD20-positive T cells were elevated in ascites fluid of epithelial ovarian carcinoma patients, whereas the percentage of CD20-positive T cells in tumor tissue and the blood of patients was not elevated^13^. CD20-positive T cells in epithelial ovarian carcinoma patients were predominantly of an EM phenotype and secreted IFN-gamma. Of note, this elevation in CD20-positive T cells in ascites fluid did not correlate with disease progression.

The origin and function of CD20-positive T cells remains a subject of debate, with one explanation being the transfer of CD20 molecules to T cells via trogocytosis during B cell/T cell interaction^13^. A second explanation is the presence of a distinct T cell subset or differentiation stage in which CD20 is transcribed in the absence of other classical B cell markers such as CD19 or immunoglobulin molecules^6^. In this respect, CD20-positive T cells are phenotypically characterized as predominant CD8 cytotoxic T cells with an EM differentiation state^5^ that produce the cytokine IFNγ^10^. In line with this EM phenotype, CD20-positive T lymphocytes were absent in cord blood samples, virtually lacking EM T cells, whereas the highest numbers were found in blood of older individuals that have a larger EM population^13,14^. CD20-positive T cells have been reported to infiltrate both primary and secondary lymphoid tissues and to have low proliferative capacity, but producing high levels of effector cytokines^3,5,6^.

To further elucidate the properties and potential function of CD20-positive T cells, we set out to identify transcriptomic differences between CD20-positive T cells and their CD20-negative counterparts from healthy volunteers by performing RNA sequencing, which revealed a reduced transmigration and an enhanced adhesive profile combined with a greater activation status. To gain insight into the functional properties of CD20-positive T cells, healthy T cells were further transduced with human CD20 cDNA to assess properties in vitro and in vivo, which revealed organ-specific reduced migratory and enhanced adhesive properties of CD20-positive T cells.

## Materials and methods

### Derivation of primary PBMCs

Three different buffy coat samples of healthy volunteers were thawed in RPMI + 10% FCS. Buffy coats were purchased from Sanquin and all donors gave informed consent (Sanquin Blood Supply, Groningen, the Netherlands). Human PBMCs were isolated via Ficoll-Paque density gradient centrifugation (Ficoll-Paque PLUS, GE Healthcare Life Sciences, Marlborough, MA, USA). Tumor infiltrating lymphocyte extraction was performed on ovarian cancer tissue obtained during surgery collected in the University Medical Center Groningen, The Netherlands. This study was carried out in the Netherlands in accordance with International Ethical and Professional Guidelines (the Declaration of Helsinki and the International Conference on Harmonization Guidelines for Good Clinical Practice. The use of anonymous rest material is regulated under the code for good clinical practice in the Netherlands and had been processed anonymously^15^. Patients had given consent to use surgical material for research purposes. Primary patient tumor infiltrating lymphocytes (TILs) used for analysis of the CD20 + TIL phenotype were isolated from fresh tumor samples obtained during cytoreductive surgery.

### Cell culture

Primary T cells, BT55 cells, CEM cells, Raji cells, HS-5 stroma cells and EBV-B cells were cultured in RPMI (Gibco-BRL, Paisly, Scotland), 10% fetal calf serum (FCS, Integro, Zaandam, the Netherlands), penicillin (100 U/ml), streptomycin (100 μg/ml) (Gibco-BRL), 5 × 10^−5 M 2-mercaptoethanol (Merck, Darmstadt, Germany). The culture medium of the primary T cells was supplemented with 300 U/ml hurIL-2. The BT55-T cell clone was restimulated once a week with irradiated autologous EBV-B cells loaded with 10 μg of CMP in a 24-wells plate. Amphotropic Phoenix cells were cultured in DMEM (Gibco-BRL), 10% FCS, penicillin, streptomycin and 2-mercaptoethanol. All cells were cultured at 37 °C in a 5% CO2 atmosphere.

### Antibodies and flow cytometry

CD20 expression was determined by mAbs specific for the human CD20 molecule conjugated with fluorescein isothiocyanate (FITC) or phycoerythrin (PE) (clone L27, BD Biosciences, San Jose, CA). The therapeutic anti-CD20 mAb was kindly provided by the pharmacy department of the University Medical Center Groningen (Groningen, Netherlands). For FACS analyses, T cells were stained with the following antibodies: CD3-PE, CD13-PE, CD4-PE, CD8-PE, CD25-PE, CD69-PE, HLA-DR-PE (BD Biosciences) and CD62L-FITC (Immunotooms). Analyses were performed on a FACS Calibur (Becton Dickinson). Rituximab FITC was generated by first performing a buffer exchange for Rituximab using Amicon Ultra-0.5 ml Centrifugal Filters for DNA and Protein Purification and Concentration (50 k, Merckmillipore) according to protocol and subsequently re-suspending it in conjugation buffer (100 mM carbonate/bicarbonate buffer, pH 9.0) at ~ 2 mg/ml. Fluorescein (FITC) (Thermo scientific) was dissolved at 1 mg/ml in DMSO. A 10 × molar excess of dissolved FITC was added to the antibody solution and mixed in the dark for 1 h at room temperature. Excess FITC was removed by performing buffer exchange using Amicon Ultra-0.5 ml Centrifugal Filters for DNA and Protein Purification and Concentration (50 k, Merckmillipore). The FITC conjugated antibody was re-suspended in PBS. An Uniform Manifold Approximation and Projection (UMAP) plot was created in FlowJo V10.5.3 (Becton, Dickinson & Company) by first down-sampling FCS files to 65,000 events and subsequently merging them before running the UMAP plugin on FlowJo V10.5.3.

### Sorting of CD20 + and CD20- CD3 + CD8 + CD45RO + CCR7-lymphocytes

Buffy coat samples were washed with PBS 2% FCS and stained for 60 min at 4 °C with Rituximab CD20-FITC, CD3-APC (eBioscience), CD8a -APC-eFluor780 (eBioscience), CD45RO- PE-Cy7 (eBioscience), CCR7-BV421 (BD), and markers CD4-PE (eBioscience), CD56-PE (eBioscience) and CD19-PE (BD). Cells were washed and filtered using a 35 µm strainer (Falcon). Propidium Iodide (1 µg/ml) was used for exclusion of dead cells. From the CD3 + CD8 + CD45RO + CCR7- population, 1000 live CD20 + and 1000 live CD20-cells were sorted using a Beckman Coulter MoFlo Astrios cytometer. UltraComp eBeads (Thermo Fisher Scientific) were used as compensation controls.

### mRNA sequencing of CD20 + and CD20- CD3 + CD8 + CD45RO + CCR7-lymphocytes

Samples were sorted in a 96-wellsplate already containing 2 µl lysis buffer (0,2% Triton X-100 (Sigma-Aldrich), 2U RNase inhibitor (Takara)) supplemented with 1 µl 10 µM oligo-dT primer and 1 µl 10 mM dNTP mix (Thermo Scientific). After sorting, the plate was spun down, incubated at 72 °C for 3 min and stored on ice. A modified SMARTseq2 protocol using custom-made primers was used (Supplementary table 1), as described previously^16,17^. In brief, SmartScribe reverse transcriptase (Westburg-Clontech) and a template switching oligo (BC-TSO) were used to generate cDNA. Next, a PCR preamplification step was done with KAPA HiFi HotStart Ready Mix (Roche Diagnostics) and a custom-made PCR primer. The cDNA samples were purified using Ampure XP beads (Beckman Coulter) in a ratio of 0,6:1 (Ampure bead: cDNA). Samples were analyzed on a 2100 Bioanalyzer using a PerkinElmer LabChip GX high-sensitivity DNA chip (Agilent) and on a Qubit™ 4 Fluorometer (ThermoFisher Scientific) according to manufacturer’s instructions. Next, 500 pg of each sample was tagmented and N7xx and S5xx index adapters were used for barcoding according to the Illumina Nextera XT DNA sample preparation kit (Illumina). Thereafter, samples were purified using Ampure XP beads (ratio 0,6 Ampure: 1 cDNA) and analyzed on a 2100 Bioanalyzer. Samples were equimolar pooled (4 nM) and subsequently sequened on an Illumina Nextseq500 2500 using 75 bp single-end reads. The obtained mRNA sequencing data was demultiplexed into individual FASTQ files followed by alignment to the human reference genome hg38 using STAR (version 2.5.2).

### RNA-seq analyses

Differentially expressed genes in CD20 + CD3 + CD8 + CD45RO + CCR7- versus CD20-CD3 + CD8 + CD45RO + CCR7-cells sorted from buffy coat samples of healthy volunteers were determined by DESeq2 (Wald test and corrected for multiple testing using the Benjamini and Hochberg method). Genes with a Benjamini–Hochberg FDR < 0.05 and log2 fold change ≥ 2 were selected for further analysis. Data was analysed using Microsoft Excel and GraphPad Prism 8. A MA-plot was generated by plotting the base mean vs the log2FoldChanges of the significant (< 0.05 p-value) genes which display at least a fold-change of 2 or higher on top of all the differentially expressed genes (DEGs). The DEGs which were found to be significantly upregulated with a fold change of ≥ 2 were tested (via Enrichr) for associations with specific ontologies based on their annotations via the Jensen TISSUES database and the KEGG 2019 human pathway (< 0.05 p-value)^18–20^. Analysis via Enrichr yielded p-values computed from a Fisher exact test. The DEGs were also analysed for enrichment using Database for Annotation, Visualization and Integrated Discovery (DAVID)^21,22^. DEGs were compared with database Tissue expression_GNF_U133A_QUARTILE (< 0.05 p-value) to annotate the gene list.

### Construction of CD20 retroviral vectors and transduction of primary human T cells

Construction of the CD20-encoding Moloney-Murine Leukemia Virus based retroviral vector and the generation of CD20 viral particles was previously described^23,24^. Primary human T cells were obtained from buffy coats of healthy donors (Bloodbank, University Medical Center Utrecht). Peripheral blood mononuclear cells (PBMCs) were centrifuged through Ficoll Hypaque (Amersham Pharmacia, Uppsala, Sweden) and stimulated with 300 U/ml human recombinant interleukin 2 (hurIL-2) (Proleukin, Chiron, Amsterdam, the Netherlands) and anti-CD3/anti-CD28 coated magnetic beads (XcyteTM Dynabeads^®^, Xcyte Therapies, Inc, Seattle, WA) for 48 h. Transductions were performed in non-treated flasks (Becton Dickinson, Kranlin Lakes, NJ) coated with 12.5 μg/ml retronectin (Takara, Otsu, Shiga, Japan). Next, after removing the beads from the cells, the cells were concentrated to 1.0 × 10^6 cells/ml and supplemented with 300 U/ml hurIl-2 and transduced with CD20 viral supernatant. After 24 h the virus supernatant was removed and the cells were resuspended in fresh culture medium supplemented with 300U/ml hurIL-2. The cells were cultured for 3–4 days and CD20 expression was determined using flow cytometry. To examine whether ectopic CD20 expression on T cells could have effect on their fitness or proliferative capacity, transduced cells were cultured for 6–12 days followed by annexin-V and PI expression analysis. Transduction with the clinical retroviral vector SFCMM-3^56,57^ was used as a control. Transduction with this vector allowed expression of the Nerve Growth Factor Receptor (NGFR) together with the herpes simplex virus thymidine kinase suicide gene. CD20-positive and NGFR-positive cells were gated to discriminate between the transduced and the non-transduced cells.

The cow milk peptide (CMP) CD4-positive clone (BT55)^25^ was stimulated with different concentration of CMP loaded onto autologous or allogeneic EBV-B cells (EBV-transformed B cells) in the presence of 50 U/ml IL-2 and 50 U/ml IL-4 (Proleukin). After 48 h cells were transduced in retronectin coated flasks with fresh viral supernatant. After 24 h the viral supernatant was removed and cells were cultured in culture medium, supplemented with 50 U/ml IL-2 and 50 U/ml IL-4. Cells were cultured for 3–4 days and CD20 expression was determined using flow cytometry.

### In vitro migration assay

The possible altered migration potential of primary human T cells from buffy coat samples ectopically expressing CD20 was examined in a trans-well system. 2 × 10^5 HS-5 cells were plated into a 24-wells plate (Corning) and incubated for 24 h at 37 °C. After 24 h, 1 × 10^5 wt T cells and T cells ectopically expressing CD20 were placed into a 5 µm trans-well plate and placed in the well containing the HS-5 stroma cells or in medium. The cells were allowed to migrate for 24 h. Cells in the bottom part were retrieved and the percentage migration through the trans-well was determined using flow cytometry based on expression of CD7 and CD13. Migration of cells in this system in the absence of stroma cells was considered to be free migration and set as 100%.

Migration in response to chemoattractants was performed with retrovirally transduced CEM-CD20 and CEM wt cells. Cells were loaded in a volume of 100 ul on transwell filters with a pore size of 5 um (Corning, Corning, NY, USA). SDF-1 (CXCL12, Immunotools GmbH, Friesoythe, Germany) was added as chemoattractant to the lower compartment at a concentration of 100 ng/mL in a total volume of 200uL of migration buffer (serum-free RPMI, 100 U/mL rhIL-2, 0.3%HSA). After 2.5 h incubation at 37 °C, cells in the lower compartment were harvested, mixed with 30 μL of counting beads (Miltenyi Biotec, Bergisch Gladbach, Germany) and quantified by flow cytometry at a fixed volume of 40 μL and high speed. The percentage of migration was calculated considering the migration of the CEM wt cells as 100%. A two-tailed unpaired t test was used to compare the levels of migration.

### IL-13 ELISA

The CMP-specific CD4-positive T cell clone (BT55) was stimulated with different concentrations of CMP (100 μg–0.001 μg) presented on autologous or allogeneic EBV-B cells for 5 h at 37 °C. 3 × 10^4 EBV-B cells were co-cultured with 3 × 10^4 BT55 cells or BT55-CD20 cells in RPMI supplemented with 10% human serum overnight at 37 °C. The supernatant was harvested and the concentration of IL-13 was determined with the Pelikine Compact human IL-13 ELISA kit according to the manufacturer’s instructions.

### Mice and conditioning regimen

RAG2-/-γc-/- mice were obtained from the Netherlands Cancer Institute (Amsterdam, The Netherlands). The mice were bred and kept in the specified pathogen-free breeding unit of the Central Animal Facility of the University of Utrecht. The animals were supplied with autoclaved sterilized food pellets and distilled water ad libitum. All animal experiments were conducted according to Institutional Guidelines after acquiring permission from the local Ethical Committee for Animal Experimentation and in accordance with current Dutch laws on Animal Experiments. The reporting in the manuscript follows the recommendations in the ARRIVE guidelines. Mice were used at 8–12 weeks of age. On day 0 mice were irradiated with a single dose of 350 cGy (TBI, 3.0 Gy X-rays) and received 0.2 ml clodronate-containing liposomes intravenously as previously described^26,27^. CD3/CD28 dynabead + IL-2 stimulated primary human T cells from buffy coat samples were transduced with a CD20 encoding retroviral vector, leading to 25% transduction efficiency (data not shown). Cells were cultured for 6 days and 40 × 10^6 cells were intravenously injected into the tail vein (n = 10). As control groups, 10 mice received 40 × 10^6 non-transduced cultured cells derived from huPBMCs and 5 mice received 15 × 10^6 fresh huPBMCs. Due to the (anticipated) development of xenogeneic graft-versus-host disease (X-GvHD), the mice were sacrificed (mice were anesthetized in a chamber containing 2.5% isoflurane in oxygen before cervical dislocation was applied), cell suspensions of the spleen were made and analysed using flow cytometry. Organs were collected from the sacrificed mice and histochemical analysis was performed to study the distribution of the injected cells. Histology was performed on spleen, lung, liver, skin, colon and bone marrow. For histology, cells were stained with the following antibodies: huCD3 (Dako cytomation: A0453) and huCD20 (Beckman Coulter, clone L26). WinMDI 2.8 software was used to analyze the CD20 expression level.

### Ethics approval and consent to participate

All blood donors gave informed consent (Sanquin Blood Supply, Groningen, the Netherlands). For TIL extraction from tumor material patients had given consent to use surgical material for research purposes. According to Dutch law no approval from our institutional review board was needed. All animal experiments were conducted according to Institutional Guidelines after acquiring permission from the local Ethical Committee for Animal Experimentation and in accordance with current Dutch laws on Animal Experiments. The reporting in the manuscript follows the recommendations in the ARRIVE guidelines.

## Results

### CD20-positive T cells predominantly display an effector memory phenotype

**Figure 1:**
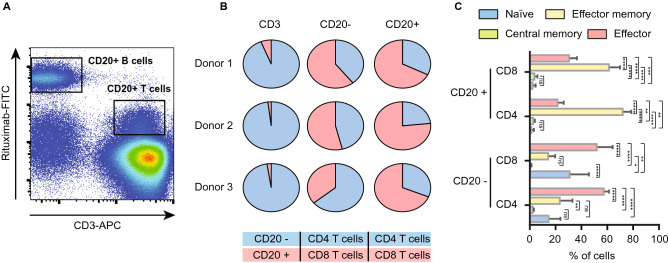
Flow cytometry analysis reveal CD20 + T cells predominantly display an effector memory phenotype. (**A**) Flow cytometry plot displaying FITC conjugated Rituximab binding on B cells and on T cells. One representative healthy donor is depicted. (**B**) Pie charts displaying CD20 + popualtion within a CD3 + population, distribution of CD4 + and CD8 + subsets within the CD20- popualtion and the distribution of CD4 + and CD8 + subsets within the CD20 + population (n = 3). (**C**) Bargraph displaying T cell differentiation status within the CD20-/CD20 + populations and within the CD4 + / CD8 + subsets (n = 3). A 2way ANOVA was performed to deterime significance.

To evaluate differences between CD20-positive T cells and their CD20-negative counterparts, the phenotype of these cell populations was first confirmed. Within the CD3-positive fraction of the PBMCs of each of the 3 healthy volunteers, a small subpopulation of CD20-dim T cells (~ 15 fold less CD20 than B cells) was detected (Fig. 1A). Whereas the CD20-negative fraction displayed a more equal distribution between CD4 and CD8 T cells (Fig. 1B), there was a significant increase in the fraction of CD8 T cells in the CD20-positive population (Fig. 1B). Further, CD20-positive T cells were significantly enriched in the CD45RO + /CCR7-EM subset in both the CD4 and the CD8 fraction (Fig. 1C). In the CD20-negative fraction this phenotype was, for both CD8 and CD4 subtypes, shifted towards an effector phenotype (CD45RO-negative and CCR7-negative) (Fig. 1C). The full gating strategy is displayed in suppl. Figure 1A. Separately, when comparing the CD8 + /CD45RO + /CCR7-/CD20 + population in the peripheral blood of healthy individuals with CD8 + /CD45RO + /CCR7-/CD20 + cells in the TIL fraction from ovarian carcinoma patients, highly similar UMAP profiles were detected (Suppl. Figure 2), highlighting the resemblance of CD20-positive TILs to peripheral blood CD20-positive T cells.

### RNAseq analysis of CD20-positive effector memory T cells reveals an altered transmigration and adhesive profile combined with an enhanced activation status

**Figure 2:**
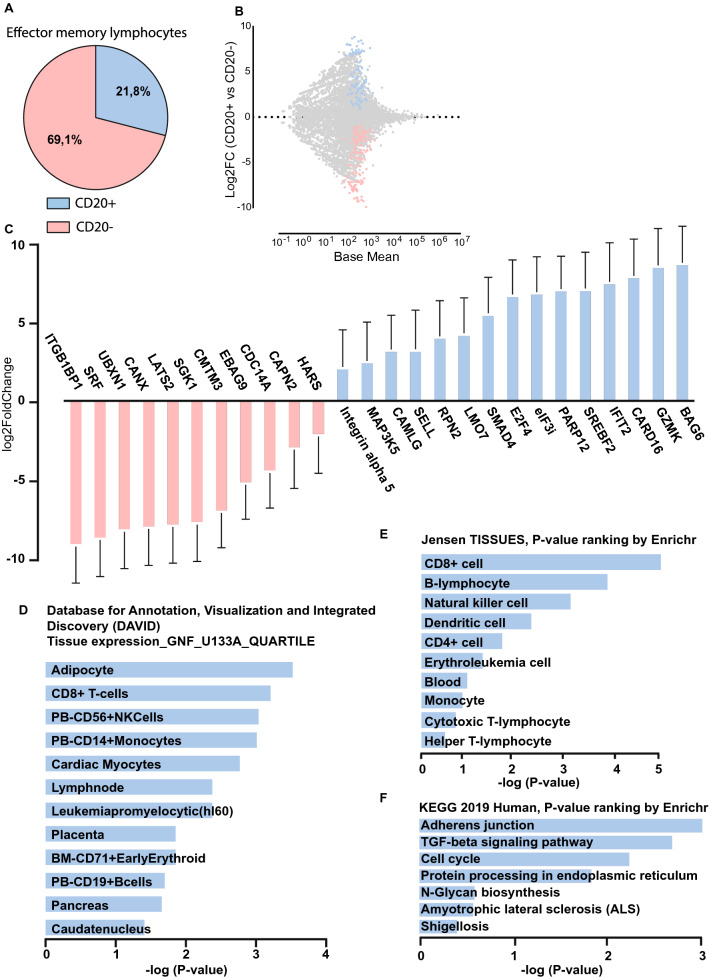
RNAseq analysis of CD20 + effector memory T cells reveals an altered transmigration and adhesive profile combined with an enhanced activation status. (**A**) Pie chart displaying FACS analysis of CD20 + T cells in a effector memory lymphocytes subset. (**B**) MA plot displaying DEGs in CD20 + CD3 + CD8 + CD45RO + CCR7- versus CD20- CD3 + CD8 + CD45RO + CCR7- with significantly upregulated genes in blue and significantly downregulated genes in red. DEGs were determined by DESeq2 (Wald test and corrected for multiple testing using the Benjamini and Hochberg method). Genes with a Benjamini–Hochberg FDR < 0.05 and log2FoldChange ≥ 2 were selected for further analysis. (**C**) log2FoldChange of a subset of significantly DEGs, with standard error (L2fcSE), with activation/memory/cytotoxic/migratory/adhesive backgrounds. (**D**) Search of DEGs for enriched terms using the tissue expression library (GNF U133A quartile) of the Database for Annotation, Visualization and Integrated Discovery (DAVID), (**E**) using the Jensen TISSUES library of Enrichr (P-value ranking) and (**F**) using the KEGG 2019 human pahtway of Enrichr (P-value ranking).

To assess the transcriptional profile, CD8 + /CD45RO + /CCR7-/CD20-positive and -negative T cells were sorted (Fig. 2A and suppl. Figure 1B for gating strategy) and analysed using RNA sequencing (Fig. 2B,C). A number of the differentially expressed genes (DEGs) found to be upregulated associate with an enhanced activation status (BAG6, CARD16, IFIT2, PARP12 and eIF3i), calcium signalling (CAMLG), effector T cell metabolism (SREBF2), memory CD8 T cell differentiation (SMAD4, E2F4, MAP3K5) or are involved in regulating leukocyte adhesion, such as GZMK, SELL, ITGA5, or LMO7. Interestingly, no upregulation of CD20 was detected within the sorted CD20-positive T cell fraction, suggesting these cells acquired their CD20 expression via trogocytosis during B cell/T cell interactions (Suppl. Figure 3A).

On the other hand, a significant downregulation was observed for ITGB1BP1 (ICAP-1), CANX (Calnexin), CMTM3 and SRF, all involved in negatively regulating cell adhesive properties. Other downregulated genes, such as HARS, UBXN1, EBAG9 and SGK1, reduce cytotoxic activity of T lymphocytes, block tumor T cell infiltration, drive T helper type 2 differentiation (TH2) or memory differentiation in CD8 T cells when expression is lost. Downregulated genes such as LATS2, CDC14A and CAPN2 are known tumor-suppressive molecules which could enhance cell cycle progression, cell mobility and reduce apoptosis. Taken together, the up- and downregulated genes in CD20-positive T cells sketch a pro-inflammatory, pro-survival, memory CD8 T cell phenotype with altered migration and adhesive properties.

By performing gene set enrichment analysis using Database for Annotation, Visualization and Integrated Discovery (DAVID), the selected DEGs were found to be enriched in database Tissue expression_GNF_U133A_QUARTILE (≤ 0.05 p-value) for several immune cells, CD8 T cells and B lymphocytes included (Fig. 2D). Using Enricher, the selected DEGs were also found to be enriched in “Jensen TISSUES” in a handful of immune-related cell types, of which CD8 T cells and B lymphocytes scored the highest based on Enricher’s p-value ranking (≤ 0.05 p-value) (Fig. 2E). Enricher also revealed enrichment in “KEGG 2019 Human”, in pathways linked to Adherens junctions and the TGF-beta signaling pathway (Fig. 2F).

### T cells ectopically expressing CD20 have normal growth properties, phenotype and response to TCR/antigen stimulation

**Figure 3:**
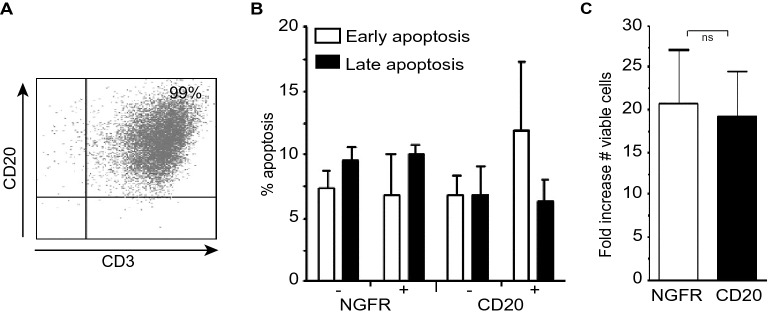
CD20-transgenic T cells display normal growth properties. (**A**) FACS-analysis of CD20 + T cells. Primary T cells were isolated from peripheral blood and transduced with a CD20-encoding retroviral vector. The transduced cells were purified by immunomagnetic cell sorting based on CD20 expression. (**B**) 1.0 × 10^6 primary T cells were transduced with the CD20 or SFCMM-3 retroviral vector (mean of 50% CD20 + or 50% NGFR-positive) and the influence of CD20 expression on the viability of cells was investigated by Annexin-V and PI staining at 7 days post transduction. Early apoptotic cells were Annexin-V + / PI- and late apoptotic / necrotic cells were both Annexin-V + / PI + . (**C**) At day 7 post transduction the absolute numbers of viable cells were determined. The bars represent the mean ± SD of three independent transductions.

To assess whether the DEGs found in CD20-positive T cells also reflect on an altered phenotypic behaviour of T cells, CD20 was ectopically expressed in T cells from healthy volunteers. After transduction and sorting, ~ 95% of the T cells co-expressed CD3 and CD20 on their surface (Fig. 3A). Ectopic expression of CD20 or transduction with an NGFR-expressing control vector did not significantly alter T cell viability, with no statistically significant difference in early apoptotic (Annexin-V-positive/PI-negative) and late apoptotic/necrotic cells (Annexin-V-positive/PI-positive) between the transduced/non-transduced NGFR-expressing control vector group and the transduced/non-transduced CD20-expressing vector group (Fig. 3B). Further, proliferation of T cells transduced with the NGFR control vector or the CD20-transduced T cells was similar, with an ~ 20-fold increase in viable cells 9 days post transduction (Fig. 3C). In conclusion, ectopic expression of CD20 in healthy T cells did not negatively impact on cell viability and proliferative capacity.

**Figure 4:**
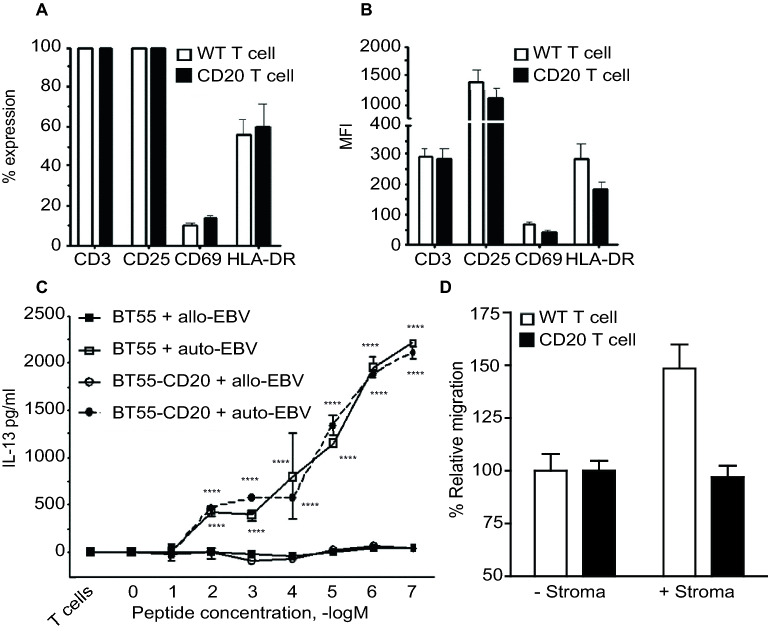
Ectopic expression of CD20 on T cells associates with altered transmigration and adhesive properties but does not alter phenotype or response to TCR/antigen stimulation. T cells were transduced with the CD20-encoding retroviral vectors (mean transduction efficiency of 50%). (**A**) CD20- and CD20 + T cells were gated and the percentage CD3, CD25, CD69 and HLA-DR and (**B**) the mean fluorescence intensity (MFI) was determined. The bars represent the mean ± SD of three independent experiments. (**C**) The cow milk peptide (CMP) CD4 + T cell clone (BT55) was transduced with the CD20-encoding retroviral vector and subsequently purified. Different concentrations of CMP (100 μg–0.001 μg) were loaded on autologous and allogeneic EBV-B cells and presented to the transduced and the non-transduced cells. IL-13 production was measured upon stimulation of the BT55 clone with increasing concentrations of CMP. The mean ± SD of three independent experiments are shown. A 2way ANOVA was performed to determine significance. (**D**) For the migration assay HS-5 stromal cells were seeded in a 24-wells plate and after 24 h of incubation at 37 °C, T cells with or without CD20 were added onto a transwell system (5 μm pore size). These transwell systems were placed into 24-wells plate containing the stromal cells or as a control the medium. After 24 h the percentage of the migrated cells was determined by FACS analyses.

To investigate whether ectopic CD20 expression altered the T cell phenotype, the expression of key T cell activation markers was evaluated, with no difference in expression of either CD25 or CD69 between the CD20-negative and the CD20-positive population (Fig. 4A). Of note, only a small portion of the cells expressed CD69 and not all cells expressed HLA-DR on their cell surface. The expression level, as determined by mean fluorescence intensity (MFI) of CD25, CD69 and HLA-DR also did not differ between the CD20-negative and CD20-positive cells (Fig. 4B).

To evaluate whether ectopic CD20 expression altered the specific antigenic response of T cells, a CD4-positive human T cell clone (BT55), which produces IL-13 upon cow's milk protein (CMP) stimulation, was engineered to ectopically express CD20. Upon loading of different concentrations of CMP (100 μg–0.001 μg) onto autologous EBV-B cells, non-transduced BT55 cells dose-dependently produced IL-13 (Fig. 4C), whereas similar stimulation with allogeneic EBV-B cells did not induce IL-13 production (Fig. 4C). Upon stimulation of CD20-positive BT55 cells with CMP-loaded autologous EBV-B cells, no significant difference in IL-13 response was detected compared to wild-type BT55 cells (Fig. 4C).

### Ectopic expression of CD20 on T cells associates with reduced transmigration

The migration potential of CD20-positive T cells in response to stromal cells was examined in a trans-well system, in which the absence of stromal cells was considered to be chemokine-independent migration and set as 100%. As shown in Fig. 4D, the presence of stromal cells enhanced the migratory potential of non-transduced T cells. However, migratory levels were reduced to those found in the absence of stromal cells upon ectopic expression of CD20 in T cells. Additionally, a reduced migratory potential towards chemoattractant SDF-1 (CXCL12) was observed for T cell line CEM ectopically expressing CD20 compared to non-transduced CEM (Suppl. Figure 3B). Interestingly, compared to non-transduced CEM, CD20-positive CEM also had enhanced expression of lymphoid homing receptor CD62L (Suppl. Figure 3C). Thus, CD20 expression on T cells affects in vitro migration in the presence of stromal cells or chemoattractants.

### CD20-positive T lymphocytes engraft and cause X-GvHD in RAG2-/-γc-/- mice.

**Figure 5:**
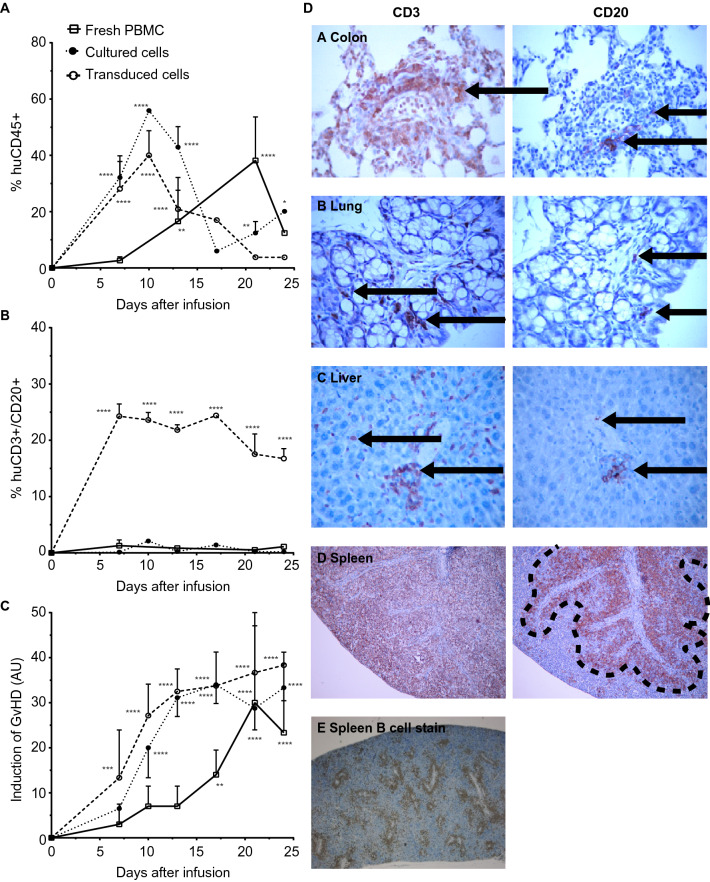
CD20 + T lymphocytes engraft, cause X-GvHD in RAG2-/-γc-/- mice and display altered adhesive pattern in mouse spleens. (**A**) 15 × 10^6 fresh huPBMCs (n = 5), 40 × 10^6 CD3/CD28 stimulated cultured primary T cells (n = 10) and 40 × 10^6 CD3/CD28 stimulated CD20-transduced primary T cells (n = 10) from the same donor were injected into irradiated and macrophage-depleted mice. The non-transduced and transduced cells were cultured for 6 days. Peripheral blood was collected once a week and the percentage of human CD45 and (**B**) CD3/CD20 + cells was determined. (**C**) The capability of the T cells to induce GvHD was determined by weight loss, reduced mobility of the mice and development of ruffled fur. These criteria were scored as arbitrary units (AU) and set against time. The mean ± SD are shown. 2way ANOVA’s were performed to deterime significance. (**D**) Immunohistochemical evaluation was performed in mice injected with T cells ectopically expressing CD20. Infiltration of human CD3 + cells is shown in the left columns and the infiltration of CD20 + cells into the organs is shown in the right columns. These figures show the colon A, the lung B, the liver C, the spleen D and spleen E (injection of non-transduced human PBMCs, anti-CD20 B cell stain).

To evaluate whether reduced transmigration of CD20-positive T cells in vitro would be recapitulated in vivo, CD20-positive T cell behaviour was evaluated in a RAG2-/-γc-/- mouse model by injection of a mixture of normal and ectopically expressing CD20-positive T cells, with a consistent average of 20% CD3 and CD20 co-expressing T cells found in the peripheral blood in time, resembling the population that was injected initially (Fig. 5A). No CD20 expression was found on cells collected from control mice injected with fresh PBMCs or T cells pre-cultured in vitro identical to CD20 expressing T cells (activation cycle prior to transduction) (Fig. 5B). From day 7 onwards, the increase in human cells was associated with an acute X-GvHD. This X-GvHD reaction was scored by characterizing weight loss, hunched posture, ruffled fur and mobility. The ectopic expression of CD20 in a subset of infused T cells did not have any impact on development and/or severity of GvHD (Fig. 5B). Flow cytometric analysis after sacrifice revealed that CD45-positive human cells were engrafted in all the treatment groups, with engraftment of CD3/CD20-positive cells being specifically detected in the spleen, as analysed in splenic cell suspensions (Fig. 5C).

### CD20-positive human T cells have an altered distribution pattern in mouse spleens

Macroscopic evaluation of histology of the colon (A), lung (B), liver (C) and the spleen (D) from mice injected with CD20-positive T cells revealed splenomegaly in all the mice as only aberrant feature, with lymphocytic infiltrates (Fig. 5D). Infiltration of CD20-positive T cells was primarily detected in the spleen, with infiltration in the lung, liver and colon being less compared to the spleen (Fig. 5DA-D, see arrows). In these organs, the CD3/CD20 T cells were diffusely infiltrated. In contrast, almost all of the CD3/CD20-positive T cells in the spleen were located in the periarteriolar lymphoid sheath (Fig. 5D, see highlighted area), whereas wild-type CD3-positive T cells were detected as fully dispersed throughout the spleen (Fig. 5DD). Interestingly, upon injection of non-transduced control human PBMCs, B cells were found to similarly locate around the arterioles (Fig. 5D,E). Thus, CD20-positive human T cells injected in mice engrafted and infiltrated into various organs. Further, the differences in distribution pattern of CD20-positive T cells compared to wild-type CD20-negative counterparts in the spleen suggest that CD20 expression on the T cells alters their homing ability.

## Discussion

In the current study, by performing RNA sequencing on CD20-positive T cells, we identified several DEGs compared to CD20-negative T cells that predominantly associated with adhesive properties of cells. Functional analysis of CD20-positive T cells revealed normal growth properties, no altered phenotype or response to TCR/antigen stimulation, but identified a reduced transmigration profile of T cells in a trans-well assay and, subsequently, in a mouse model. Specifically, ectopic expression of CD20 on T cells triggered preferential localization in the periarteriolar lymphoid sheath in a pattern similar to that of human B cells. In the other organs analysed, there was no difference in infiltration between CD20-positive T cells compared to wild-type CD20-negative counterparts. This suggests that CD20 expression on T cells alters the homing ability in the spleen. Altogether this study revealed an altered transmigration and adhesive profile for CD20-positive T cells.

One of the notable differences at the messenger RNA level in CD20-positive T cells is a set of genes involved in adhesion/migration. Specifically, various significantly upregulated genes (e.g., GZMK, Integrin alpha 5, LMO7 and SELL) all are reported as positive regulators of leukocyte adhesion. GZMK is involved in trans-endothelial diapedesis, the process where leukocytes exit the blood circulation and enter sites of inflammation or tissue injury^19^. GZMK is enhanced in EM T cells and on the transcriptomic level EM T cells with high GZMK expression display characteristics of Th17 cells, which has previously been linked to the CD20-positive T cell subset in patients with RA^8^. By RT-qPCR, upregulation of GZMK was confirmed within the CD20-positive fractions of each donor (Suppl. Figure 3A). Expression of integrin alpha 5, another cell adhesion molecule, is increased in memory T cells and is a known facilitator of initial extravasation of leukocytes from the circulation into inflamed tissue^28^. LMO7 is shown to interact with nectin–afadin and E-cadherin-catenin systems (cell–cell adhesion molecules) and therefore could be an important player in the adhesive properties of CD20-positive T cells^29^. Especially upregulation of SELL, also known as L-selectin or CD62L, is of interest, as this protein is described as a major player in leukocyte adhesion, migration and signalling and is also used to distinguish between central memory (TCM, CD62L + CCR7 +) and EM (TEM, CD62L − CCR7 −)^30,31^. Interaction of peripheral node addressin, the ligand for SELL, which is found on high endothelial venules (HEVs), with its receptor SELL, mediates leukocyte rolling and facilitates extravasation into secondary and tertiary lymphoid organs^32,33^. Lymphocytes of mice harbouring a mutant form of SELL are unable to bind to peripheral lymph node HEV^34^. SELL expression has been linked to CD8 memory T cells residing mostly in the T cell zone around the arteriole supply of the spleen, whereas SELL-negative cells were predominantly found in the red pulp. It was also demonstrated that upon antigen re-challenge these memory cells could rapidly proliferate and subsequently the secondary CD8 effector T cells were found in the red pulp^35,36^. Enhanced expression of CD62L was confirmed in CD20-positive CEM (Suppl. Figure 3C). Curiously, although within the gene set enrichment analysis association with B lymphocyte fractions was detected, no upregulation of CD20 was identified within the sorted CD20-positive T cell fractions, suggesting these cells acquired their CD20 expression via trogocytosis during B cell/T cell interactions as previously reported^13^.

On the other hand, several inhibitors of adhesion were found to be downregulated. For instance, ITGB1BP1 (ICAP-1) expression in CD20-positive T cells, which suppresses integrin activation and blocks cell adhesion^37,38^. An additional down-regulated gene was CANX (Calnexin), a chaperone protein involved in cellular adhesion, with knock-out of CANX upregulating adhesion molecules VCAM1 and ICAM1^39^. In addition, CANX impairs CD4 and CD8 T cell activity by inhibiting proliferation and reducing secretion of IFNγ, TNFα, and IL2^40^. Another significantly downregulated gene, CMTM3, is directly involved in VE-cadherin turnover, with CMTM3 knockdown inhibiting VE-cadherin internalization^41^. CMTM3 also has tumor suppressive functions, inhibits proliferation and migration^41^. SRF, serum response factor, is described to, via miR-199a-5p transactivation, inhibit E-cadherin and is therefore linked to gastric cancer metastasis^42^. Two other significantly downregulated genes, HARS and EBAG9, reduce cytotoxic activity of T lymphocytes and block tumor T cell infiltration^43–45^. Collectively, the findings on the DEGs involved in cell adhesion in CD20-positive T cells align with the experimental findings of reduced migration in the migration assay and the accumulation of CD20-positive T cells in the periarteriolar lymphoid sheath in the mouse model with ectopically expressing CD20-positive T cells.

In line with literature on CD20-positive T cells, in which these cells are described as IFNγ producing, CD8 cytotoxic T cells with an EM differentiation state^5,10^, RNA sequencing of CD20-positive T cells revealed upregulation of BAG6, CARD16, IFIT2, PARP12 and eIF3i, which could be responsible for the enhanced inflammatory phenotype often associated with CD20-positive T cells^46–50^. We also identified a significantly upregulated gene being directly involved in enhanced calcium signalling, CAMLG^51^. Moreover, a significant upregulation of genes involved in in the acquisition of effector T cell metabolism (SREBF2)^52^ or genes (SMAD4, E2F4, MAP3K5) involved in differentiation into memory CD8 T cells was detected^53–55^. Although both CD4 and CD8 T cell populations were observed within the CD20-positive T cell fraction, also without an effector memory phenotype, we only sorted and analysed the CD20-positive and CD20-negative CD3 + CD8 + CD45RO + CCR7- lymphocytes, as this was the enriched phenotype observed.

Subsequent ectopic expression of CD20 using a retroviral vector led to efficient expression of the complete CD20 molecule, which did not alter the growth characteristics or spontaneous apoptosis levels of T cells. Further, CD20 expression did not impact on response upon TCR/antigen stimulation. These findings are in contrast with other literature on CD20-positive T cells in which it is stated that these cells display a significantly lower proliferation rate and a higher susceptibility to apoptosis compared to CD20-negative T cells^3^. These differences might have arisen from the dissimilarities between physiological and ectopic CD20 expression. Interestingly, in our study, ectopic CD20 expression in primary T cells and T cell line CEM did impair in vitro migration in the presence of stromal cells or in response to chemoattractant SDF-1 (CXCL12). This finding fits with a report on B cells in which it is described that whereas CD20 expression is dispensable for B cell receptor signalling, it is suggested to be indispensable for actin cytoskeleton polymerization, cell adhesion and migration^56^. Further, upon CD20 knock-out, most B cell lines became unresponsive to homeostatic chemokines such as SDF1α, CCL19 and CCL21. Reversely, and in line with our findings, B cell line Ramos actually displayed an enhanced migratory profile in response to homeostatic chemokines when CD20 was knocked out. The differences between these B cell lines might be due to the fact that MEC1 cells originate from chronic B cell leukemia while the Ramos cell line represents Burkitt lymphoma, with over 4000 DEGs between these two cell lines^56^. In future studies the impact of ectopic CD20 expression specifically on CD4 and CD8 T cell subsets should be assessed, as here, although both CD4 and CD8 subsets were present, we merely examined the impact of ectopic CD20 expression on the whole T cell population.

Further, a mix of normal and ectopically expressing CD20-positive T cells induced X-GvHD in mice in the same degree as normal T cells alone, confirming normal T cell behaviour in vivo upon ectopic CD20 expression. Interestingly, immunohistochemical analysis of mouse organs after sacrifice demonstrated a similar degree of T cell infiltration in the majority of the organs analysed, with the notable exception of the spleen. Within the spleen, CD20-positive T cells located specifically in the periarteriolar lymphoid sheath, similar to B cells and similar to previous reports in which SELL expression has been linked to CD8 memory T cells residing mostly in the T cell zone around the arteriole supply of the spleen^36^. Positioning of B and T cells is achieved by differential expression of chemokine receptors on the lymphocytes and adhesion molecules expressed by the endothelial cell of the HEVs in different anatomical compartments in secondary lymphoid organs^57^. The differences in distribution pattern of the CD20-positive T cells compared to their CD20-negative counterparts in the spleen suggest that CD20 expression on B cells and T cells affects the homing abilities and the lymphoid positioning of these cells.

In conclusion, our data collectively point to unique phenotypical and behavioural properties of CD20-positive T cells that predominantly impact on transmigration and adhesive properties, but also on activation and memory status. This study further delineates the physiological properties of this T cell subset present in both physiological and pathophysiological conditions and paves the way further delineation of the (patho)physiological role of CD20-positive T cells.

## Supplementary Information

Supplementary Information 1.

Supplementary Information 2.

Supplementary Information 3.

Supplementary Information 4.

## Data Availability

The accession number for the sequencing data reported in this study is GSE184833.

## Abbreviations

NGFR: Nerve growth factor receptor
EM: Effector memory
RA: Rheumatoid arthritis
MS: Multiple sclerosis
DEG: Differentially expressed gene
PBMCs: Peripheral blood mononuclear cells
CMP: Cow milk peptide
FITC: Fluorescein isothiocyanate
X-GvHD: Xenogeneic graft-versus-host disease
TIL: Tumor infiltrating lymphocyte
HEVs: High endothelial venules
