# Supplementary material for: CD20 positive CD8 T cells are a unique and transcriptionally-distinct subset of T cells with distinct transmigration properties
Source: Sci Rep. 2021 Oct 15;11:20499. doi: 10.1038/s41598-021-00007-0 (PMC8520003; doi:10.1038/s41598-021-00007-0)
Supplement: Supplementary file 1 — Supplementary Information 1. [file 41598_2021_7_MOESM1_ESM.pdf]

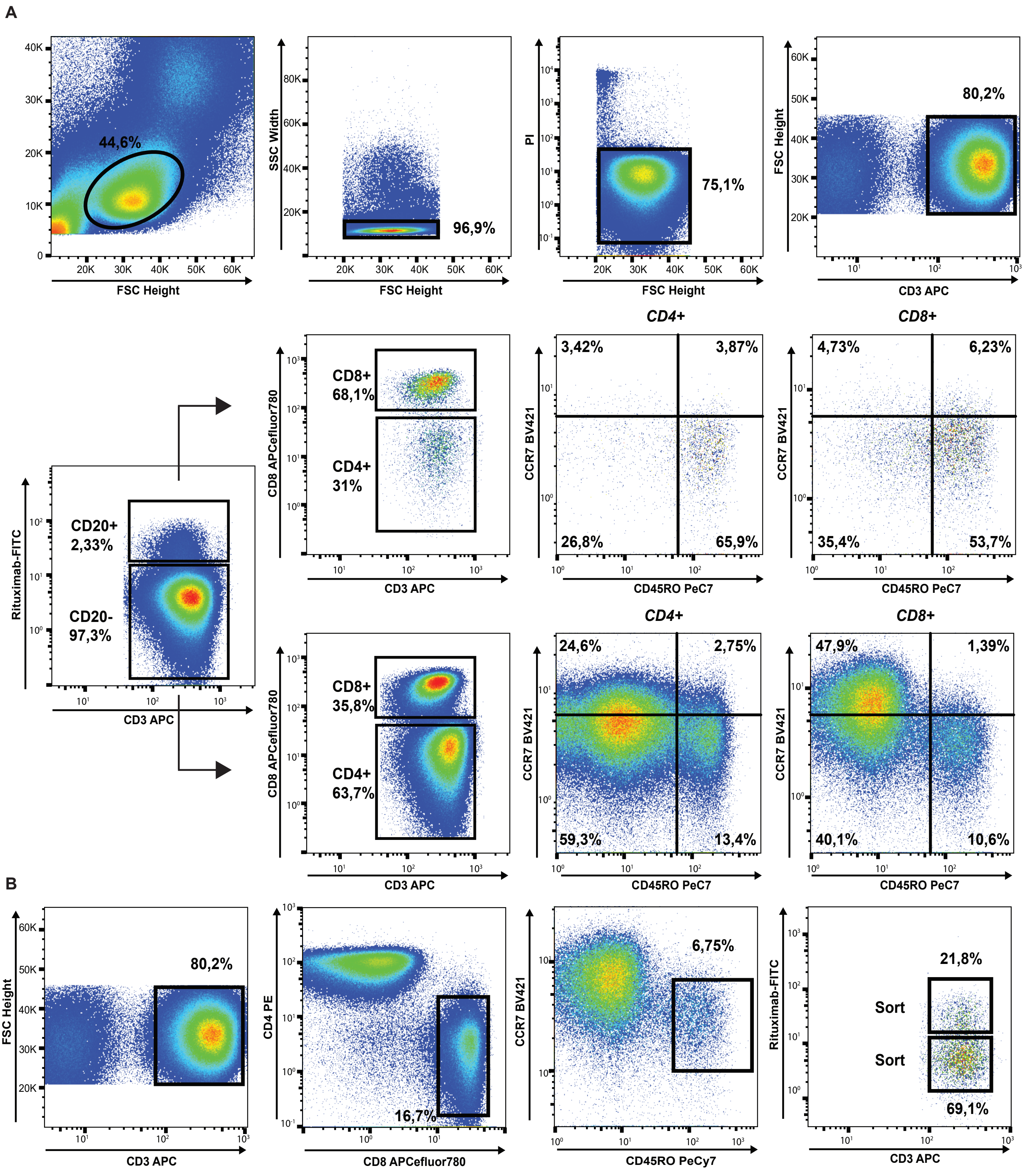

Supplemental Figure 1. Gating strategy for sorting and phenotyping of CD20-positive T cells.
A FACS plots dipicting the gating strategy for phenotyping of CD20+ and CD20- lymphocytes. **B** Gating strategy for sorting CD20+ and CD20- effector memory lymphocytes.
