# Supplementary material for: CD20 positive CD8 T cells are a unique and transcriptionally-distinct subset of T cells with distinct transmigration properties
Source: Sci Rep. 2021 Oct 15;11:20499. doi: 10.1038/s41598-021-00007-0 (PMC8520003; doi:10.1038/s41598-021-00007-0)
Supplement: Supplementary file 2 — Supplementary Information 2. [file 41598_2021_7_MOESM2_ESM.pdf]

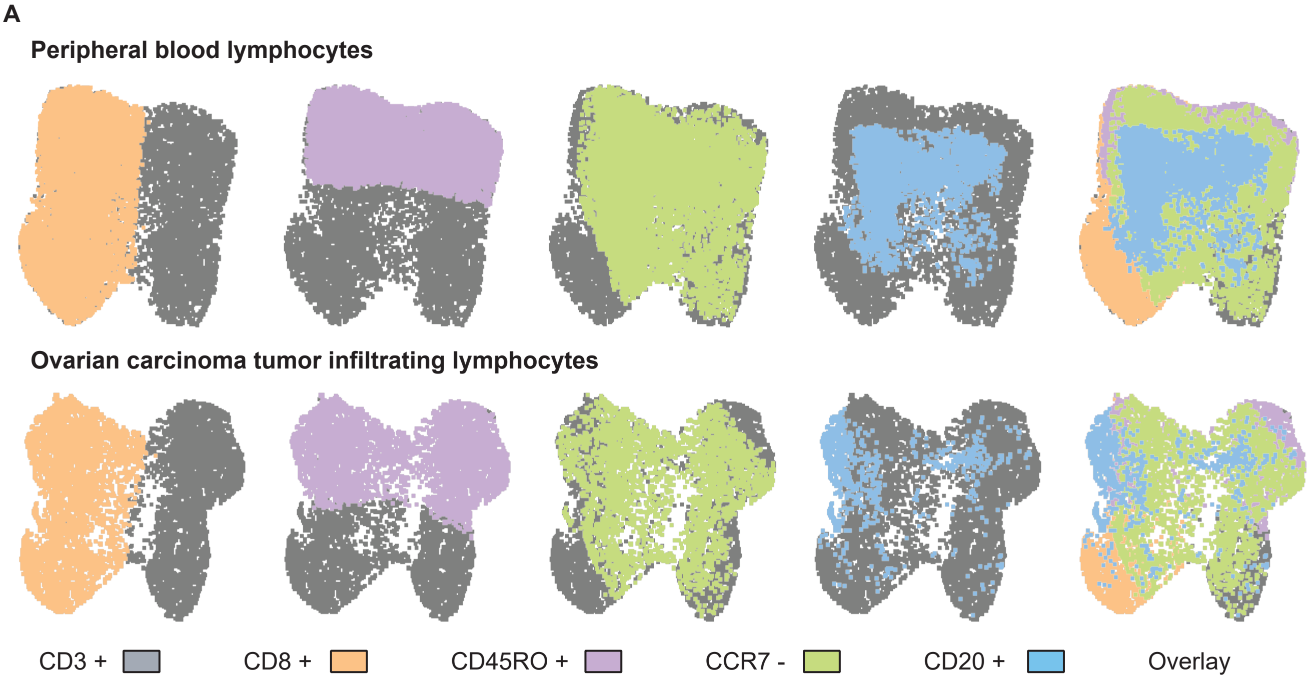

Supplemental Figure 2. Resemblance of CD20-positive TILs to peripheral blood CD20-positive T cells.
A UMAP representation of CD3+/CD8+/CD45RO+/CCR7-/CD20+ cells in the peripheral blood and in an ovarian carcinoma tumor infiltrating lymphocyte population isolated from fresh tumor samples obtained during cytoreductive surgery (N=3).
