# Supplementary material for: CD20 positive CD8 T cells are a unique and transcriptionally-distinct subset of T cells with distinct transmigration properties
Source: Sci Rep. 2021 Oct 15;11:20499. doi: 10.1038/s41598-021-00007-0 (PMC8520003; doi:10.1038/s41598-021-00007-0)
Supplement: Supplementary file 3 — Supplementary Information 3. [file 41598_2021_7_MOESM3_ESM.pdf]

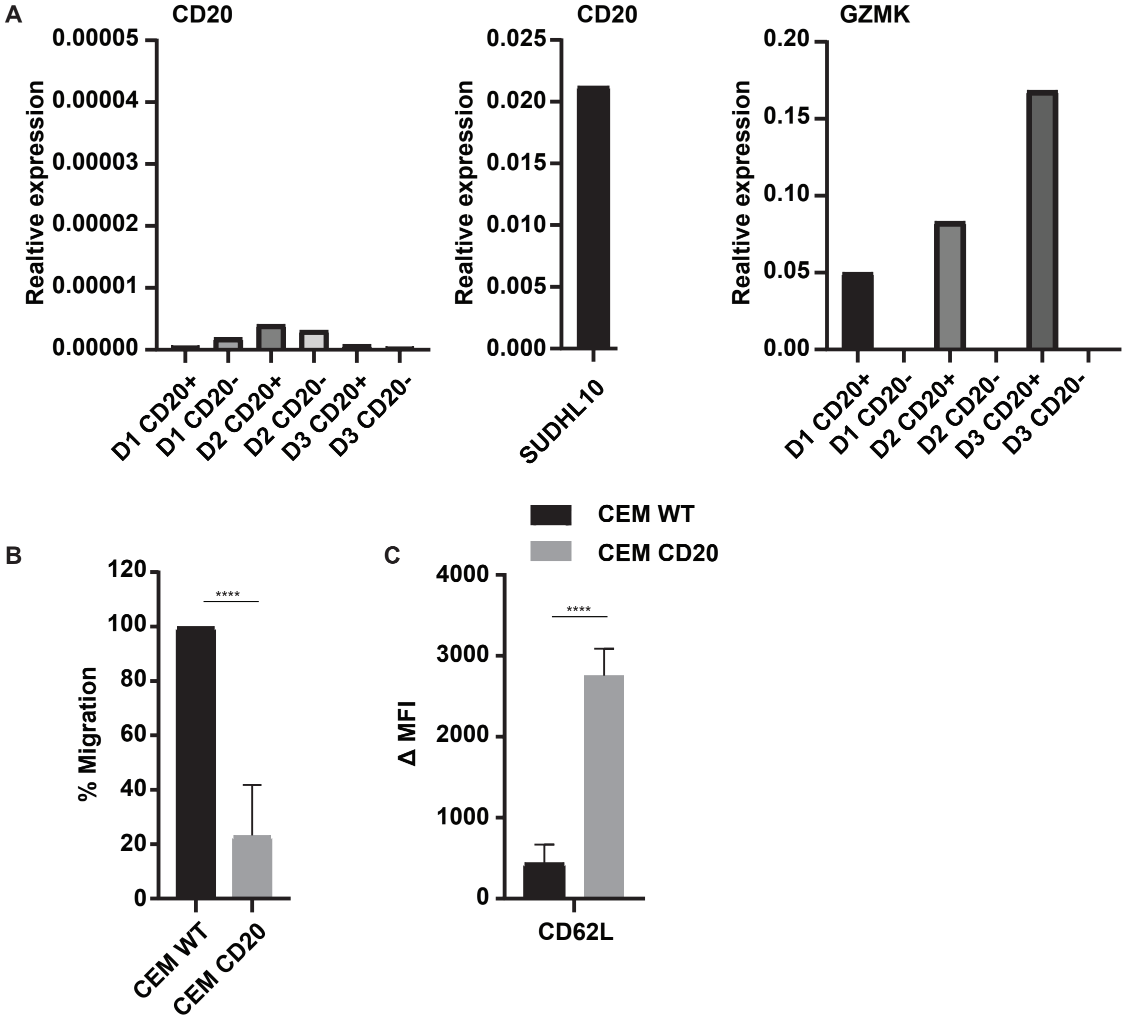

Supplemental Figure 3. RT-qPCR validation of RNA-Seq data.
A RT-qPCR analysis of CD20 (left) and GZMK (right) from the 3 individual donors (CD20+ and CD20- fractions) used in the RNA-Seq analysis. B cell line SUDHL10 was taken along as positve control for the expression of CD20 (middle). 2^-dCT's are displayed on the y-axis. **B** Migration in response to chemoattractants was performed with retrovirally transduced CEM-CD20 and CEM wt cells. CEM-CD20 and CEM wt cells were loaded in a volume of 100 ul on transwell filters with a pore size of 5 um. SDF-1 (CXCL12) was added as chemoattractant to the lower compartment at a concentration of 100 ng/mL in a total volume of 200uL of migration buffer. After 2.5 h incubation at 37°C, cells in the lower compartment were harvested and quantified by flow cytometry. The percentage of migration was calculated considering as 100% of migration the migration of the CEM wt cells. A two-tailed unpaired t test was used to compare the levels of migration. **C** Expression of lymphoid homing receptor CD62L on CEM wt and CEM-CD20 determined by flow cytometry. An unpaired t test was used to compare the expression levels.
