# Supplementary material for: CD20 positive CD8 T cells are a unique and transcriptionally-distinct subset of T cells with distinct transmigration properties
Source: Sci Rep. 2021 Oct 15;11:20499. doi: 10.1038/s41598-021-00007-0 (PMC8520003; doi:10.1038/s41598-021-00007-0)
Supplement: Supplementary file 4 — Supplementary Information 4. [file 41598_2021_7_MOESM4_ESM.pdf]

Primer	sequence
BC-Oligo-dT	5'- AAG CAG TGG TAT CAA CGC AGA GTA CTT TTT TTT TTT TTT TTT TTT TTT TTT TTT TTT TCA-3'
BC-TSO	5'- AAG CAG TGG TAT CAA CGC AGA GTG AAT RGR G+G-3'
BC-PCR	5'- AAG CAG TGG TAT CAA CGC AGA GT-3'

Nextera index 1 (i7) adapters (N7)

index name	i7 bases in adapter
**N701**	TCGCCTTA
**N702**	CTAGTACG
**N703**	TTCTGCCT
**N704**	GCTCAGGA
**N705**	AGGAGTCC
**N706**	CATGCCTA

Nextera index 2 (i5) adapters (S5)

index name	i5 bases in adapter
**S502**	CTCTCTAT
**S503**	TATCCTCT
**S504**	AGAGTAGA
**S517**	GCGTAAGA

RNA solution (cells are sorted in this solution):

	volume
cell lysis buffer (0,2% Triton and 2U / ul RNAse inhibitor)	2 ul
10 uM oligo dT primer	1 ul
4x 10 mM dNTP mix	1 ul
	tot 4 ul

Reverse transcription mix:

	volume	Final concentration
Smart scribe reverse transcription (100 U/ul)	0,5	5 U / ul
Rnase inhibitor (40 U/ul)	0,25	1 U / ul
smart scribe first strand buffer (5x)	2	1x
DTT (100 mM)	0,2	2 mM
Betaine (5M)	2	1 M
BC-TSO (10 uM)	0,1	1 uM
Nuclease free water	0,95	
	tot 6 ul (end volume = 10 ul)	

Reverse transcription program:

Cycle	Temp	Time	Purpose
1	42	90 min	RT and template-switching
2-11	50	2 min	unfolding of RNA secondary structure
	42	2 min	Completion/continuation of RT and temlate-switching
12	70	15 min	enzyme inactivation
13	4	hold	safe storage

PCR preamplification mix:

	volume	Final concentration
KAPA HiFi HotStart ReadyMix (2x)	12,5 ul	1x
BC-PCR primer (10 uM)	0,25 ul	100 nM
Nuclease free water	2,25 ul	
	tot 15 ul (end volume = 25 ul)	

PCR preamplification program:

Cycle	Temp	Time
1	98	3 min
2-24	98	20 sec
	67	15 sec
	72	6 min
25	72	5 min
26	4	hold

Supplementary Table 1. Sample preparation, Primers and PCR protocol for mRNA sequencing.
Preparation of mRNA sequencing samples, primer sequences and the PCR protocol used. Tagmentation according to Illumina nextera protocol.
